# A large pericardial cyst mimicking a unilateral pleural effusion: A case report

**DOI:** 10.1097/MD.0000000000033540

**Published:** 2023-04-14

**Authors:** Minfang Li, Chunfeng Yang, Jinshuai Li, Dan Jia, Yaqiong Wang, Wei Xie, Jinlin Wang

**Affiliations:** a Department of Respiratory Medicine, Shenzhen Traditional Chinese Medicine Hospital, Shenzhen, China; b The Second School of Clinical Medical Sciences, Guangzhou University of Chinese Medicine, Guangzhou, China; c The Fourth Clinical Medical College of Guangzhou University of Traditional Chinese Medicine, Guangzhou, China; d Department of Respiratory Disease, The State Key Laboratory of Respiratory Disease, China Clinical Research Centre for Respiratory Disease, Guangzhou Institute of Respiratory Health, First Affiliated Hospital of Guangzhou Medical University, Guangzhou, China.

**Keywords:** pericardial cyst, pleural effusion, VATS

## Abstract

Pericardial cysts are a rare benign disorder with a variable clinical presentation depending on their size and location. The diagnosis of pericardial cysts is usually based on imaging examinations. The definitive treatment is surgical resection.

**Patient concern:**

A 36-year-old woman presented with progressive left-sided chest pain and exertional dyspnea, with symptoms resembling pleural effusion.

**Diagnoses:**

The patient was diagnosed with a pericardial cyst based on imaging and video-assisted thoracoscopic surgery (VATS).

**Intervention:**

VATS was performed.

**Outcomes:**

The patient’s symptoms improved after successful removal of the pericardial cyst. Follow-up chest computed tomography exhibited no evidence of recurrence.

**Lessons:**

Clinicians should include pericardial cysts in the differential diagnosis of pseudopleural effusion. VATS is a feasible and safe method to treat symptomatic and large pericardial cysts.

## 1. Introduction

Pericardial cysts are rare and benign congenital malformations with a variable clinical presentation, and these mediastinal abnormalities occur in 1 person per 100,000.^[1]^ More than 50% of such patients have no symptoms, but the cysts are occasionally revealed by routine chest radiological examinations.^[2]^ The exact clinical presentation and complications depend on the size, location of the cyst and presence of involvement/invasion into adjacent structures.^[3]^ We present an interesting case of a woman with a large pericardial cyst in an uncommon location, mimicking unilateral pleural effusion. Informed consent was obtained from the patient and her family.

## 2. Case presentation

**Figure 1.**
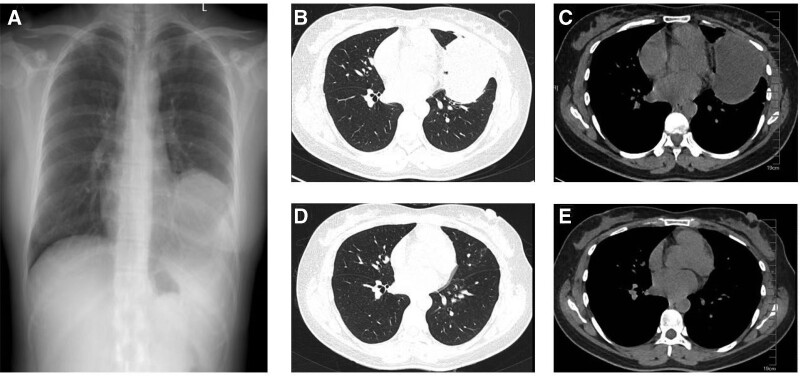
(A–C) CT scans of the chest showed multiple encapsulated effusions adjacent to the left ventricle in the left costophrenic angle. (D and E) Follow-up chest CT scans. CT = computed tomography.

**Figure 2.**
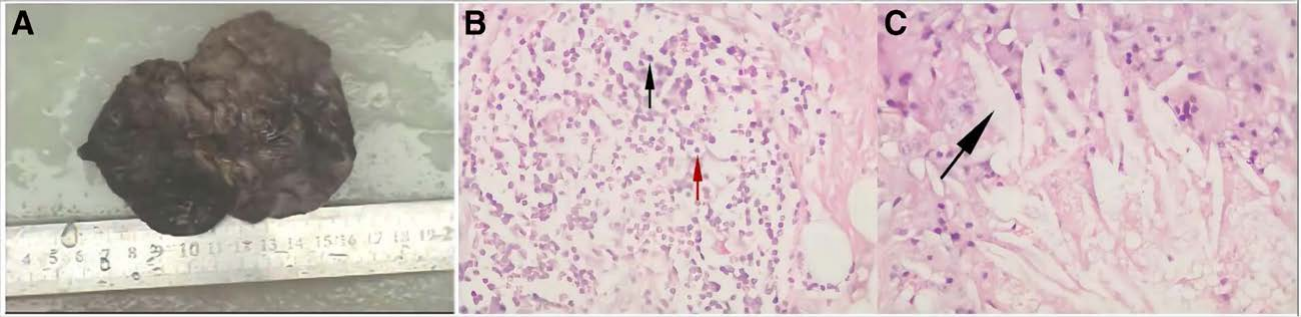
(A) Macroscopic appearance of a giant pericardial cyst measuring 10.5 cm × 2.5 cm × 2 cm in size. (B) Histopathologic examination revealed collagen and scattered elastic fibers with a thin mesothelial lining and chronic inflammation (red arrow: lymphocyte; black arrow: plasmocyte), confirming the diagnosis of an inflamed pericardial cyst. (C) Cholesterol crystal deposition (arrow).

A 36-year-old woman presented with progressive left-sided chest pain and exertional dyspnea for 4 months. Before being referred to our institution, she had been treated with thoracentesis and an indwelling pleural catheter, with a total of 2500 mL of drainage from the effusion. Diagnostic antituberculosis treatment was offered, but no response was observed. Her medical history was unremarkable except for chest trauma from a fall while riding a bicycle 10 years ago. The physical examination was remarkable for decreased breath sounds with dullness to percussion on the left. Further electrocardiographic tests and laboratory investigations were mostly negative. A chest computed tomography (CT) scan showed loculated left-sided pleural effusion (Fig. 1). A small amount of brown-colored transudative fluid was collected by ultrasound-guided left thoracentesis. Biochemical, microbiological and cytological analyses of the pleural fluid were unremarkable. Percutaneous needle aspiration and pleural biopsy demonstrated punctured striated muscle and fibrous tissue without granuloma or malignant cells. Further drainage of the effusion was unsuccessful. For further investigation, we performed video-assisted thoracoscopic surgery (VATS) and found that the previously supposed loculated effusion was a pericardial cyst, which was filled with brown fluid, measured 10.5 cm × 2.5 cm × 2 cm in size, originated toward the lateral aspect of the pericardium near the left ventricle, and adhered to the lung and pleura. Histological examination confirmed the diagnosis of an inflamed pericardial cyst (Fig. 2). After successful removal of the pericardial cyst, the patient’s symptoms improved. Follow-up chest CT performed 6 months after discharge exhibited no evidence of recurrence.

## 3. Discussion

The clinical presentation of pericardial cysts varies. The common symptoms are chest pain, cough, dyspnea, and cardiac arrhythmias. Some serious complications, including sudden death, cyst rupture, atrial fibrillation, and pericarditis, have been reported in patients with large pericardial cysts or those in unusual sites.^[4]^ In our case, the symptoms of chest pain and dyspnea were caused by compression of the surrounding thoracic structures due to the uncommon size of the cyst. No demonstrable compression of the left ventricle was observed.

The most common radiographic appearance of pericardial cysts is a well-defined, unilocular or multilocular round or oval mass with smooth walls and a diameter ranging from 1 to 5 cm.^[5]^ Seventy percent of the cases have been reported to be located in the right cardiophrenic angle, with 22% in the left cardiophrenic angle, and 8% in the anterior-superior or posterior part of the mediastinum.^[6,7]^ However, the diagnosis of pericardial cysts can be especially challenging due to their rarity and is more difficult when the lesion occurs outside the typical location, as in this case. Furthermore, when the lesion is extensive and involves mediastinal and thoracic structures, the atypical presentation further complicates the diagnosis.^[8]^ In this case, this symptomatic giant pericardial cyst was initially incorrectly diagnosed as encapsulated pleural effusion in the left chest due to the lesion appearing to be in contact with the pleura and left lower lung. The nonspecific fluid density of the cyst also posed a diagnostic challenge in distinguishing it from loculated effusion on imaging. In addition, the content of the cyst appeared to be thick brown fluid, which was different from the commonly described “springwater cysts” with presence of crystal-clear fluid within the pericardial cyst. To improve the diagnostic accuracy for pericardial cysts, the understanding and diagnostic awareness of pericardial cysts needs to be improved.

The most common etiology of pericardial cysts is congenital causes due to failed fusion of mesenchymal lacunae during the embryogenic state.^[1,9]^ However, other causes of pericardial cysts have also been described in the literature, including inflammation (rheumatic pericarditis, bacterial infection particularly tuberculosis, echinococcosis), trauma, complications of cardiac surgery and chronic hemodialysis.^[10]^ The etiology of this case may be congenital, and the pericardial cyst was discovered in middle age during the 4th decade of life, which is consistent with the majority of cases reported in previous literature.^[10]^ However, our patient had a definite history of chest trauma 10 years ago, so a traumatic origin could not be excluded.

Pericardial cysts are usually discovered and monitored by radiological modalities. They are usually revealed as an incidental finding during chest X-ray scans carried out on asymptomatic patients. Transthoracic echocardiography provides the exact location and characteristics of the lesion, providing better recognition.^[11]^ CT and magnetic resonance imaging are helpful in showing detailed information on the density of the mass, anatomical location of the pericardial cyst and the association with surrounding structures and in differentiating cysts from other malformations, such as chest malignancies.^[5,12]^ However, each imaging modality has its own limitations, and histological examination is warranted to obtain a consolidated diagnosis and established a definite diagnosis.^[8]^

The need for changes in management depends on the patients’ symptoms and complications and the size and location of the cyst.^[13]^ In most patients, close follow-up with serial transthoracic echocardiography is sufficient for monitoring and to ensure a benign course in which the pericardial cyst can resolve spontaneously.^[11]^ Other treatments include percutaneous aspiration, surgical intervention with VATS or surgical excision.^[5,14]^ Our patient’s cyst was large enough to cause symptoms, so surgical intervention was necessary. Initially, we tried to perform percutaneous aspiration for further workup to establish the diagnosis, but this approach was unsuccessful. The histochemical examination of the fluid from the cyst and aspiration biopsy was unhelpful and nonspecific for diagnosis. Finally, the definitive diagnosis was confirmed by VATS, and the pericardial lesion was removed. Therefore, VATS is a feasible and promising method in symptomatic patients with cardiorespiratory repercussions, as described in the present case.

## 4. Conclusions

We reported a rare case of a large pericardial cyst located in an uncommon location mimicking unilateral pleural effusion and finally confirmed the diagnosis and achieved resolution through VATS. Therefore, clinicians should include pericardial cysts in the differential diagnosis of pseudopleural effusions. VATS is a feasible and safe method to treat symptomatic and large pericardial cysts.

## Author contributions

**Conceptualization:** Minfang Li, Wei Xie, Jinlin Wang.

**Formal analysis:** Minfang Li, Jinlin Wang.

**Funding acquisition:** Wei Xie.

**Investigation:** Minfang Li, Chunfeng Yang, Jinshuai Li, Dan Jia, Yaqiong Wang, Wei Xie, Jinlin Wang.

**Methodology:** Minfang Li, Dan Jia, Jinlin Wang.

**Validation:** Minfang Li, Jinlin Wang.

**Visualization:** Minfang Li, Wei Xie, Jinlin Wang.

**Writing – original draft:** Minfang Li, Jinshuai Li, Dan Jia, Yaqiong Wang, Wei Xie, Jinlin Wang.

**Writing – review & editing:** Minfang Li, Chunfeng Yang, Dan Jia, Yaqiong Wang, Wei Xie, Jinlin Wang.
